# The financial transaction between counseling and nursing care service centers (CNCSCs) and their clients: a qualitative study

**DOI:** 10.1186/s12913-018-2934-z

**Published:** 2018-04-12

**Authors:** Sefollah Alaei, Fatemeh Alhani, Hassan Navipour

**Affiliations:** Department of Nursing, Faculty of Medical Sciences, Tarbiat Modares University, Jalal Al-Ahmad, P.O. Box 14115-331, Tehran, Iran

**Keywords:** Counseling and nursing care service centers (CNCSCs), Qualitative research, Financial transaction with the clients, Financial management

## Abstract

**Background:**

Community-oriented nursing care is an important model of nursing care. Counseling and Nursing Care Service Centers (CNCSCs) have been providing these private services to the Iranian community for nearly two decades. Resource management, cost-benefit analysis and affordability are important steps in providing these services. The present study was conducted to explore the challenges of financial transactions between CNCSCs and their clients.

**Methods:**

This study has a qualitative design and was conducted on a total of 30 participants, consisting of CNCSC managers, staff, physicians and clients who were selected through purposive theoretical sampling. Data were collected through in-depth interviews and direct observations and were analyzed using conventional qualitative content analysis.

**Results:**

The analysis of the data led to the extraction of three main categories, including the flaunted atmosphere due to direct financial transaction, instability in determining tariffs for nursing services and the use of strategies for cost-effective services and client satisfaction.

**Conclusion:**

To increase affordability and satisfaction and expand private community-based nursing.

Services, appropriate financial policies should be designed and applied that can lead to transparent and simple financial transactions with the clients by way of indirect monetary exchanges. These policies should be designed in a systematic manner with integrity, facilitate inter-sectorial cooperation in the health sector and be cost-effective for the clients, insurance companies and the health system.

**Electronic supplementary material:**

The online version of this article (10.1186/s12913-018-2934-z) contains supplementary material, which is available to authorized users.

## Background

In line with the WHO objectives for Primary Health Care (PHC) [1, 2] and the Comprehensive Health.

Action Plan 2013–2020 [3], health policies have been established to create a balance between different levels of prevention and to provide community-based services [4].In some countries, some basic changes in health system policies have made family and community-based services grow to the level that they now account for half of all the health services provided to the public, and the amount of health services provided by hospitals is now equal to the amount of services given by the community [5].

The activities of Counseling and Nursing Care Service Centers (CNCSCs) in Iran can be considered a logical step in promoting nurses’ professional role in the community [6].These private centers are allowed to offer different nursing services to individuals, families and the community; however, the majority of their services are currently offered at homes. Community-based nursing care has a longer history in countries such as the United States and Canada, where these centers are supported by the local health systems and have a major share in the delivery of health services [7–9]. In developing countries the role of nurses in community-based healthcare provision is ambiguous [10–12] and nursing care is mostly restricted to hospitals [13–15].

Community-based health services offer several advantages, such as saving in costs [16, 17]. A major challenge for health system managers is to improve the quality of healthcare at the same time as reducing its costs [18]. The costs of providing health care are always increasing [19] and the need to increase the budget allocated to the health sector and design efficient input-output evaluation mechanisms for improving the efficiency of health financing [20] are more evident than ever. Performing cost-benefits and cost-effectiveness analyses and adjusting reimbursement policies can accelerate the process of expanding community-based health service provision [21, 22].

Particularly in developing countries, health systems are faced with the serious challenge of resource management [23] and healthcare delivery [24, 25]. At present, the privatization of health care services might be a step too far in assisting the health system to face its challenges [26]; nonetheless, this step is being taken in developing countries without a clear and coherent strategy [27].

Modern approaches to health care have identified three main goals for an ideal health care system, which include quality improvement, accountability and fair financial contribution to health care services [28, 29]. From the clients’ perspective, the most important attributes of good health care services are their easy accessibility, cost-effectiveness, affordability and high quality [30]. In addition, health care organizations and community-based health care centers need to have an acceptable level of income in order to be able to continue providing their services.

Financial profit is naturally a priority of private nursing services, even with the governmental support offered to these centers in developed countries, financing the services was a main concern [7, 31]. Few studies have examined the economic aspect of community-based nursing care [22]. Identifying the challenges in the financial management of nursing services can help policymakers and CNCSC managers design appropriate strategies for expanding community-based nursing services.

Having a payment schedule and providing cost-effective services are important for service providers, clients and other stakeholders in any financial affair. In addition to the price of the commodities or services, how and when the costs are calculated and paid and what documentations are needed for these financial transactions is also key. In other words, the mean of financial transaction means how to calculate the cost of services, how and when should to pay and which documentation is required.

In spite of nearly two decades of activity, the services provided by CNCSCs are not well-organized and are faced with great financial problems. The present study was conducted to explore the financial transaction between CNCSCs and their clients and the challenges in their management and the contributing factors.

## Methods

The present qualitative study was conducted using the content analysis method. The main participants included CNCSC managers, but the data obtained made the researchers recruit physicians and clients too. Sampling continued until data saturation was achieved with 28participants and complementary interviews were conducted with two other family members(Table 1).The inclusion criterion was to have at least one year of work experience(in the past or the present) for having sufficient experience in a CNCSC and the exclusion criterion was unwillingness to participate in the study.

**Table 1:** Participants’ demographic characteristics

Participants	Sex	Age (years)	Work experience (years)	Educational level
NCSC managers 11	Man19	37–67	1–35	PHD 4
Nurse 8	Woman 11			Bachelor’s degree: 18
nursing assistant 3				Master’s degree student: 4
inspector of NCCC 1				Diploma 4
family member 3				
Physician 4				

Data collection began with in-depth individual interviews, and the researcher tried to gather complementary data through the observation of the financial transactions made in the centers and their related documents, if possible. The main interview questions were‘ Please discuss your experiences of communicating with clients and providing care to them’ and ‘Please discuss your experiences of establishing a financial relationship with your clients, the estimated costs of the services, the tariffs and their payment by the clients’(Additional File 1: interview guides).

Before each interview, the researchers introduced themselves to the interviewees and briefed them on the study objectives and methods and ensured them of the confidentiality of their data and that they would not be used against them. The participants then signed informed written consent forms for participation in the study. The recorded interviews were transcribed verbatim. Data were analyzed using the conventional content analysis, in which pre-existing theories had no place and where data analysis was based on the meanings that the data conveyed. In this approach to data analysis, the researcher repeatedly peruses the collected data in order to obtain a general understanding of the subject [32]. The data obtained in the study were analyzed through the following steps: Preparing the data, defining the unit of analysis, developing codes and categories and a sample text, encoding the entire body of text, ensuring consistency in encoding, drawing conclusions from the encoded data, and reporting the findings [33].

The trustworthiness of the data was ensured through different strategies, such as the allocation of adequate time to the research, holding in-depth interviews, explaining the objectives of the study to the participants in detail and performing a member check and a peer review [34].

## Results

The findings of the study led to the extraction of three main categories, including the flaunted atmosphere due to direct financial transaction, instability in determining tariffs for nursing services and the use of strategies for cost-effective services and client satisfaction. This section explains the main categories and subcategories (Table 2).

**Table 2:** Exploring the challenges in the financial transaction between CNCSCs and their clients

Main Category	Subcategory	Initial Code
A**:**Theflaunted atmosphere due to direct financial transaction	A1. Direct unfavorable monetary exchanges	The financial pressure on clients caused by their direct payment of the costs incurred by services
		Problems in the reimbursement of the costs due to the lack of public insurance coverage
		The reluctance and inability to direct out of pocket payment for health-related expenses
	A2. The complex interaction with private insurance companies	The low acceptance of CNCSCs and the independent rule-setting by some private insurance companies
		The complex documentation needed for the reimbursement of costs by complementary insurance
		The incomplete reimbursement of costs by private insurance companies
B:Instability in determining tariffs for nursing services	B1.Inadequate attention to CNCSC services	The ambiguity and undefined roles of CNCSCs in price-setting
		Delay in updating Irrational and outdated prices
	B2.The need to bargain with the CNCSCs and clients to fix a price	Bilateral (CNCSC-client) efforts for financial profiting
		Clients being uncertain about the price of nursing services offered at CNCSCs
	B3. A defective environment of competence	Independent nursing home care by nurses and clinics
		Easier access to inexpensive, although unprofessional and low-quality, home care services
		The client’s preference for receiving services from hospitals and governmental health care centers given their comparatively lower prices
C: The use of benefit strategies for cost-effective services and client satisfaction	C1. Regulating financial transactions with the families	Financial transactions limited to only one of the family members
		Avoiding a direct financial transaction between the CNCSCs staffs and the family members
	C2. Expediency trying to expand organizational relationships	A cautious work relationship with some semi-private organizations
		Trying to expand professional relationships with rich and financially-independent organizations
		Trying to expand the delivery of services to clients with complementary insurance coverage
	C3.Trying to rationalize the costs of service for the clients	Informing the clients about the sensitivity and benefit of community-based nursing services
		Assisting the clients in preparing adequate documentation for the reimbursement of costs by complementary insurance

### A. The flaunted atmosphere due to direct financial transaction

This category consisted of two subcategories, including direct unfavorable monetary exchanges and the complex interaction with private insurance companies.

#### Direct unfavorable monetary exchanges

Most clients have to personally pay for CNCSC services without their public insurance plans covering their associated costs. Even when the cost of their desired service is reasonable, they have to pay it entirely out of pocket (OOPE) and as soon as they receive the service; as a result, the whole scenario still seems exorbitant, especially compared to physicians’ fees or public hospitals’ and clinics’ fees that are covered by insurance.

*“When we refer patients to CNCSCs for continuing their care, many of them or their families come and complain about the high direct costs of these services. For example, they pay200,000 IRR for a physician’s visit, but have to pay 400,000 IRR for a wound dressing!”(P.10- General Surgeon).*

Another reason extracted was that the clients of these services and their families did not understand the importance of nursing care. Their poor understanding of the scientific nature of nursing and community-based nursing care affected their willingness to pay for nursing services. In some cases, due to insufficient family income and the costs of multiple illnesses, they were not able to pay additional fees for nursing care services too.

*“Things such as injections are viewed as very simple tasks by most people and they pay less attention to the consequences the nurses may face if they don’t properly perform them, so, they are often reluctant to pay for these services. Sometimes, they don’t even have the money to pay for them at all” (P.8- CNCSC Manager).*

#### The complex interaction with private insurance companies

Some private insurance (PI) companies do not accept the bills issued by CNCSCs or insist on having them separately confirmed by physicians.

*“I feel that private insurances do not accept nursing as an independent profession. At CNCSCs, we charge a fee for wound dressing care and say that the physician should confirm it too, even though wound care is a separate nursing duty”(P.22- CNCSC Manager).*

In some cases, PI contracts cover services through the complementary insurance (CI) cards they issue to the insured person. Through the card, the client can receive health services free of charge or pay a small part of the bill out of pocket (OOP), which is the main advantage of health insurance for clients. Sometimes, however, due to the poor interaction between CNCSCs and PI companies or the delays in reimbursement of the fees by the PIs, CNCSCs prefer to be paid for the cost of their services directly and therefore bill the clients on the spot. This form of financial transaction is considered arduous and undesirable for the clients.

*“I have been using CNCSC services for a long time to care for my elderly and sick parents, but since the beginning of this year, they (CNCSC) began asking me to pay the costs out of pocket and billed me for different things. It has gotten complicated and takes a lot of time to prepare the documentation now. What triggered this was that the PI companies delayed reimbursing the CNCSCs. The financial part became very difficult and I decided to stop getting services from CNCSCs”(P. 27- Family Member).*

### B. Instability in determining tariffs for nursing services

This category consisted of three subcategories, including inadequate attention to CNCSC services, the need to bargain with the CNCSCs and clients to fix a price, and a defective environment of competence.

#### Inadequate attention to CNCSC services

Poor price-setting for nursing services are another important barrier to effective financial transaction with the clients. Most of the participants said that the prices of most care services they offered in the centers were either still not fixed or were not up-to-date. For example, although preventive education and counseling have been defined as the main role of these centers, they lack a clear pricing and are not very cost-beneficial and there has been very little public interest in these forms of assistance. The sole recipients of education and counseling are the patients or their family members, who are eager to learn more about self-care and the steps of referring their patient to a physician or hospital.

*“We offer advice and assistance to our patients and do all of this alongside our other care services …” (P. 4 and P6- CNCSC Nurses).*

#### The need to bargain with the CNCSCs and clients to fix a price

By not setting or updating the prices, CNCSC managers have to constantly negotiate with their clients to reach an agreement about the price of the requested services or else may have to use prices set by other clinics or healthcare providers.

#### A defective environment of competence

Inadequate supervision by the health system, inadequate familiarity with CNCSCs and financial problems are some of the challenges faced by these centers. Moreover, some hospital nurses take the liberty of providing home care to patients without having the required certificates.

*“Sometimes, the hospital nurses establish a certain relationship with their patients and their family that may lead to non-regulated home nursing care and so we lose some of our clients” (P. 17- CNCSC Manager).*

Some healthcare clinics also provide home care in spite of the legal constraints. Some people may even be receiving nursing services by non-professionals and unlicensed health providers, as the clients sometimes prefer inexpensive albeit low-quality services over costly but high-quality professional nursing services.

*“Say, a patient comes to our center for urethral catheterization and asks about its costs. I hold a degree, and my response is, 600.000 IRR. A non-professional health worker may suggest 350.000 IRR for the same job. Patients do not notice that the worker who suggests 350.000 has no expertise in performing catheterization. Unfortunately, the patients often go to lay health workers who ask 350.000 for the job. So, you end up seeing the patient refer to our center with a severe urinary tract infection and many other problems” (P. 6- CNCSC Manager).*

Meanwhile, many of the services provided by CNCSCs have become more accessible at lower prices in public hospitals and clinics.

### C: The use of benefit strategies for cost-effective services and client satisfaction

This category consisted of three subcategories, including regulating financial transactions with the families, expediency trying to expand organizational relationships, and trying to rationalize the costs of service for the clients.

#### Regulating financial transactions with the families

Patients or elders who need care are often in a poor physical and mental status and cannot properly participate in the decision-making regarding their own care. To create transparency in the contracts, management and monitoring of care services and to have financial discipline in the calculation of prices and their payment, CNCSC managers prefer to have their professional relationship and financial transactions with one of the family members throughout the process of service delivery.

*“Even if this older person or patient has children and they are willing to participate in the care and payment process, we draw up our contract with only one of them”(P. 7- CNCSC Manager).*

Meanwhile, CNCSCs prohibit financial transaction between the CNCSC nurses and the patients or their families.

*“One of the caregivers I sent to someone’s home was taking money from the family. I got very upset, but the family said he was doing a really good job. I said it isn't acceptable for us that they get any money from you because they may develop a habit of it. So, I dismissed that caregiver, after all”(P. 12- CNCSC Manager).*

#### Expediency trying to expand organizational relationships

Some private and semi-private organizations such as banks and municipalities have independent insurance plans for their employees and their families. These organizations are willing to contract with CNCSCs for receiving nursing services, but tend to propose lower prices. With such contracts, CNCSCs sometimes have to provide services in locations that are far from their main office, and this long commute increases the time spent and the costs of the services and essentially reduces the cost effectiveness of the services. CNCSC are therefore cautious in concluding contracts with these organizations.

*“Several bank authorities visited our center and agreed to contract with us. They even drafted the contract, but then I thought it may not be cost-effective for us to offer them these services at these prices and so decided not to work with them” (P. 5- CNCSC Manager).*

Nonetheless, there have been successful instances of service centers establishing work relationships with some private and semi-private organizations with good financial capacities.

#### Trying to rationalize the costs of service for the clients

To increase the affordability of CNCSC nursing services, these centers try to make families understand the importance and sensitivity of community-based nursing services. They brief the patients and their families on the benefits of receiving services from these centers, such as the less commute to healthcare centers and reduced costs along with the improved quality of services. These explanations demonstrate the superiority of CNCSC nursing services over non-professional and cheaper services.

*“We try to make the family understand their mistakes… That if they spend100.000 IRR less, it may not really be better for the patient, as they may develop complications and receive a very poor-quality service” (P. 15- CNCSC Supervisor).*

The complicated process of getting reimbursed by complementary insurance companies makes the clients have to ask CNCSCs to help them with getting the appropriate documentation and perhaps building an informal relationship with the physicians to have their bills confirmed.

*“We take the invoices to specialists to confirm, and this is only possible through informal relationship with the doctors, because it is not truly their job” (P. 3 and p.22.CNCSC Managers).*

## Discussion

The findings revealed the flaunted atmosphere due to direct financial transaction and instability in determining tariffs for nursing services as factors increasing annoyance, confusion, out-of-pocket payment and client dissatisfaction. To reduce the negative effect of these factors on the affordability of the services, CNCSC managers usually use cost-benefit strategies for offering cost-effective services and increasing client satisfaction.

Public insurance plans do not cover the costs of CNCSC services. The inadequate insurance coverage for nursing services negatively affects the financial transaction between nursing care service providers and clients in CNCSCs, increases OOPs and ultimately reduces the use of community-based nursing services. Clients have to pay the entire costs of using these services themselves and may have to pay other costs, simultaneously and therefore experience great financial difficulties. The results of other studies also suggest that the multiplicity of payments for health care services complicates financial transactions and has negative effects on financial management [35]. The poor insurance coverage offered for nursing services means that even when the services are offered at a fair price, some families find them expensive and unaffordable, especially given that they may be able to receive similar services at a lower cost in public hospitals and clinics.

The increased (CI) coverage by (PI) companies can be considered an opportunity for private health care providers such as CNCSCs. Nonetheless, the relationship between CNCSCs and these companies is gradually growing and some of the barriers, such as not having a clear and customized policy for community-based nursing services, may be resolved. It should be noted that CI helps those of the community who are insured voluntarily (rather than obligatory through their employee) who often are relatively in better financial situation and can pay CIs a premium [36, 37]. Therefore unlike in advanced countries [38, 39], low-income peoples benefit from CNCSC services to a lower degree. Like many other studies [40, 41], the present study found that the inadequate insurance coverage for nursing services imposes a great financial burden on families and makes them reluctant to seek such services. The heavy costs of healthcare can limit the clients’ access to high-quality and cost-effective healthcare services [19]. Lack of health insurance in developing countries may mean enormous OOPs [42, 43], while countries with better healthcare systems rely less on OOPs [44–46]. Nursing centers in these countries have a flourishing market and their policies are such that reimbursement for these services is adequate both by public and private insurance companies [31].

There are three main healthcare delivery models, including public assistance, health insurance and national health services. The health authorities in Iran adopted the Public Assistance model [47] and fund it in a pluralistic way through the social security organization [25] and by way of an annual government health budget [44], taxes, social security insurance payments and out-of-pocket payments [26, 46]. In this model, health decision-making, planning, resource management and service delivery fall under the responsibility of the government [47]. Consequently, government policies and plans can dramatically affect the presence and activities of healthcare delivery centers, including CNCSCs. The negative impact of policies counteracts and weakens the health management process [48].

Like in other developing countries, Iran’s health care system is faced with complexities in marketing and management [25, 49], such as the overuse of health care services [26] and insufficient funding (41). Such challenges have imposed serious limitations on the efficiency, quality and equity of the healthcare services [26]. The role of health insurance is sub-optimal from the perspective of health insurance organizations, health care providers and clients [25]. In spite of the significant increase in public insurance coverage in the recent decade [26] and the health transformation program in place [50], Iran’s health system has not been successful in reducing OOPs in community-based health centers significantly [51].

According to the present study, the absence of clear tariffs and an agreement on CNCSC services confuses the clients about the real costs of the services. Other studies have similarly shown that clients may pay different fees for a similar service and some insurance plans do not cover the costs of these services and direct payments increase drastically [45]. Due to the underestimations about the importance of community-based nursing care, most patients and families are reluctant to pay for nursing services. The heavy costs of these services may mean that the patients suffice to lower-quality care from informal service providers. The clients’ willingness to pay is an important component of the cost-benefit analysis of health services [22, 52]. Nursing has a long history in developed countries and community-based nursing care is very extensively offered; people may also have ample knowledge about the practice of nursing in these countries and might truly appreciate its benefits and thus put their trust in nurses [53]. In developing countries, however, the novelty of community-based nursing services means that more serious attempts are needed to gain a wider public interest. Increasing the variety of nursing services offered can help the public better perceive the nurses’ role and their capabilities and thus change the attitude toward nursing services and nurses [54].

The present study showed that public health centers and hospitals in Iran do not collaborate with CNCSCs and have almost no interaction with these centers. Unlike these findings, other studies have shown a good relationship between community-based nursing care centers and the good accessibility of their services [31]. As a result, people are rarely informed about or referred to these centers in Iran. The poor integrity of health system programs and the weak interactions between the private and public health sector [55].The lack of economic expertise in health care management, the poor monitoring of the services and OOPs are other challenges faced with the privatization of health care [25].Improving financial management skills in healthcare managers is therefore as vital as it is in nursing managers [38, 56].

The conditions of care delivery and the financial transactions between Iranian CNCSCs and their clients are remarkably similar to home-based nursing practices in Turkey. Most Turkish people cannot use home-based nursing services due to the inadequate insurance coverage offered for these services [57]. In countries such as the United States and Canada, however, most nursing centers are supported by comprehensive healthcare plans such as Medicare or receive financial support from the government, and most people, even low-income groups, can widely benefit from these services [7, 53, 58].

The three main stakeholders in a health-related financial transaction are the clients, the service providers (CNCSCs) and the insurance organization. Some important points should be taken into account when establishing such financial transaction: 1. The price of the services should be clear and fair; 2. All the stakeholders should achieve a fair profit by engaging in this transaction, 3. Processing monetary payments should be carried out with clarity and simplicity, and 4. Every health care program must have a systematic design and application. The cost-benefit analysis of health care services should consider the costs and the direct and indirect benefits of these services [59]. Failing to conduct a financial analysis of programs increases the final costs of health care services [40].

In spite of the greater willingness toward indirect payment and the separation of payment from service delivery [40], the present study showed that the financial transaction between CNCSCs and their client move toward direct payments. The low insurance coverage’s, the direct payments with complex and low reimbursements and the heavy out-of-pocket payments for CNCSC services have created serious challenges in the affordability and expansion of these private community-based nursing care centers (Additional File 2: FigureS1). Fixing a minimum/maximum time allowed for payment sand finding the best methods of payment are necessary for private health care delivery management [60].

CNCSC managers use certain strategies to offer cost-effective services and increase their clients’ satisfaction. They enter agreements with semi-private organizations with great financial resources that request various nursing services and try to convince the clients about the benefits of receiving CNCSC services and paying their costs and help them provide appropriate documentation for CI reimbursement by PIs. Nonetheless, some of their actions, such as forcing the clients to make direct payments, are cause for client dissatisfaction.

Iran’s Ministry of Health has well understood the challenges and seeks to up-to-date the tariffs of some of CNCSC services and improves the professional interaction between hospitals and these centers. To date, these efforts have not yielded practical results in terms of increasing the insurance coverage for these services and the problems persist, such as direct payments, high OOPs, poor affordability of the services and increased client dissatisfaction and confusion.

The stability and development of private community-based nursing services rely on cooperation between CNCSCs, clients, the health system and insurance organizations. In addition to the health system, private community-based service centers such as CNCSCs should also seek to develop policies and plans that take account of the benefits of the main stakeholders and thus improve the quality of health services and decrease their costs to a more reasonable level. At the same time, CNCSCs must convince health systems and insurance companies that the expansion of their activity reduces the health system burdens and the costs of public and private insurances.

### Limitations

Given the private nature of CNCSC services, they are less obliged to collect and keep their financial documents than formal public health organizations. They can even hide their real incomes and expenses. The researcher was not able to access the CNCSCs’ financial documents, especially their income forms.

Given the limited number of studies on community-based nursing service centers, future studies are recommended to further explore the other aspects of these centers.

## Conclusion

Insufficient public insurance coverage and the lack of access to an up-to-date price list increase direct payments and lead to confusion and drive away the clients and thus reduce the use of CNCSC services.

To increase the affordability of these services, increase client satisfaction and expand private community-based nursing care, financial policies should be designed to enable clear and simple financial transactions with the clients through indirect payments, increased insurance coverage and reduced out-of-pocket payments. These policies should be designed with integrity and in a systematic manner and should facilitate cooperation between different sectors of the health system and lead to cost-effective services for the clients, insurance companies and the entire health system.

## Additional files

Additional file 1: Interview guides of CNCSCs financial transaction with clients. Brief description of the data: Interview guides that developed specifically and used in research process in research entitled: "The financial transaction between Counseling and Nursing Care Service Centers (CNCSCs) and their clients: A qualitative study" (DOCX 15 kb)

Additional file 2: **Figure S1.** Influencing factors on CNCSCs financial transaction with clients. Brief description of the data: This file Designed for better clarify effective factors on CNCSCs financial transaction with clients and strategies that to be applied with CNCSCs managers. (DOCX 34 kb)

Additional file 3: Raw data of CNCSCs financial transaction with clients. Brief description of the data: Raw data which gathered in research process about CNCSCs financial transaction with clients (DOCX 23 kb)

## Abbreviations

CI: Complementary Insurance
CNCSCs: Counseling and Nursing Care Service Centers
IRR: Iranian Currency (Rial)
OOPs: Out-Of-Pocket payments
PI: Private Insurance
