# Supplementary material for: The financial transaction between counseling and nursing care service centers (CNCSCs) and their clients: a qualitative study
Source: BMC Health Serv Res. 2018 Apr 12;18:282. doi: 10.1186/s12913-018-2934-z (PMC5898021; doi:10.1186/s12913-018-2934-z)
Supplement: Supplementary file 1 — Interview guides of CNCSCs financial transaction with clients. Brief description of the data: Interview guides that developed specifically and used in research process in research entitled: "The financial transaction between Counseling and Nursing Care Service Centers (CNCSCs) and their clients: A qualitative study" (DOCX 15 kb) [file 12913_2018_2934_MOESM1_ESM.docx]

**The financial transaction between Counseling and Nursing Care Service Centers**

**(CNCSCs) and their clients: A qualitative study**

Pre Question “warm-up” question:

- Please tell me about yours primary motivating for setting up the CNCSC?
- Can you explain me about kind of healthcare that you gave to clients in your center?
- Please explain about managerial activity in a working day

Main Question:

- Please discuss your experiences of communicating with clients and providing care to them
- Please discuss your experiences of establishing a financial relationship with your clients
- Please explain about how estimating costs (tariffs) of the services.
- Please explain your experience about how receiving or payment the services cost.

After analyses data guided us to interview with client, we focused on this these questions from clients:

- Please explain your experience about relationship and contract with CNCSC
- Please explain your experience about how receiving or payment the services cost.

To clarify the participants’ experience, Probing questions were also asked based on the data provided, for example:

- I’m not quite sure I understood …Could you tell me about that some more?
- Can you tell me more about that?
- What would that look like?
- How do you do that?

Post (final) Question:

- Thank you for all that valuable information, is there anything else you’d like to say to me?
