# Supplementary material for: The financial transaction between counseling and nursing care service centers (CNCSCs) and their clients: a qualitative study
Source: BMC Health Serv Res. 2018 Apr 12;18:282. doi: 10.1186/s12913-018-2934-z (PMC5898021; doi:10.1186/s12913-018-2934-z)
Supplement: Supplementary file 2 — Figure S1. Influencing factors on CNCSCs financial transaction with clients. Brief description of the data: This file Designed for better clarify effective factors on CNCSCs financial transaction with clients and strategies that to be applied with CNCSCs managers. (DOCX 34 kb) [file 12913_2018_2934_MOESM2_ESM.docx]

| **High** direct financial transaction led to **decrease** tendency to CNCSCs services  **Low** direct financial transaction led to **increase** tendency to CNCSCs services  **Public Health service (Hospital, clinics)**  Strong relationship  Poor relationship  No Relationship  **Public Health Insurance**  **Private Insurance**  **Health system** |
| --- |
