# Supplementary material for: The financial transaction between counseling and nursing care service centers (CNCSCs) and their clients: a qualitative study
Source: BMC Health Serv Res. 2018 Apr 12;18:282. doi: 10.1186/s12913-018-2934-z (PMC5898021; doi:10.1186/s12913-018-2934-z)
Supplement: Supplementary file 3 — Raw data of CNCSCs financial transaction with clients. Brief description of the data: Raw data which gathered in research process about CNCSCs financial transaction with clients (DOCX 23 kb) [file 12913_2018_2934_MOESM3_ESM.docx]

**Table2. Exploring the challenges in the financial transaction between CNCSCs and their clients**

| **Main Category** | **Subcategory** | **Initial Code** | **Primary(Raw data)** |
| --- | --- | --- | --- |
| A**:The flaunted atmosphere due to direct financial transaction** | A1. **Direct unfavorable monetary exchanges** | The financial pressure on clients caused by their direct payment of the costs incurred by services | Direct payment of the total cost of services by the client due to not coverage of general insurance with CNCSCs services |
|  |  |  | Patient and family complaints of high directly paying cost of CNCSCs services |
|  |  |  | Total high cost of home care than hospitalization for clients due to continues care needs |
|  |  |  | Financial strain caused by the cost of counseling services and other costs of illness |
|  |  |  | Annoyance and lack of referral to the counseling center due to costs |
|  |  |  | The focus of the patient and the family is on direct costs payment rather than focusing on overall benefits |
|  |  | Problems in the reimbursement of the costs due to the lack of public insurance coverage | Lack of adequate compensation mechanism of reimbursement of CNCSCs services clients |
|  |  |  | The severity of the services costs payment for clients due to the non-return of payment |
|  |  | The reluctance and inability to direct out of pocket payment for health-related expenses | The low tendency of the community to pay for health services costs |
|  |  |  | Lack of proper understanding of clients about importance nursing home care. |
|  |  |  | Lack of patient and family interest in receiving services from the center due to the high and direct payment costs of services |
|  |  |  | The peoples reluctance to pay for health care services |
|  |  |  | The lack of proper payment due to the Oversimplification of nursing services. |
|  |  |  | The inability of some low income families to pay for CNCSCs services costs |
|  | A2. **The complex interaction with private insurance companies** | The low acceptance of CNCSCs and the independent rule-setting by some private insurance companies | Disapproval of some of the service cost fees by of some of the CI providing companies |
|  |  |  | Avoid certain PI companies to direct contracts with CNCSCs. |
|  |  |  | unclear policies of PI companies in establishing a working relationship with counseling centers |
|  |  | The complex documentation needed for the reimbursement of costs by complementary insurance | Complex and time-consuming provision of documents to provide insurance companies with supplementary services coverage |
|  |  |  | The insistence of some PI to seize the invoices by doctors |
|  |  |  | clients request to receive an invoice for a higher amount to compensate for the cost difference |
|  |  | The incomplete reimbursement of costs by private insurance companies | OOP paying for part of the services costs due to lower proposed tariffs by PI in compare with the CNCSCs tariffs. |
|  |  |  | Delay in repayment by PI for CNCSCs services costs |
|  |  |  | Directly Paying of costs CNCSCs and delay in repayment by PI. |

**Table2. Exploring the challenges in the financial transaction between CNCSCs and their clients**

| **Main Category** | **Subcategory** | **Initial Code** | **Primary(Raw data)** |
| --- | --- | --- | --- |
| B:**Instability in determining tariffs for nursing services** | **B1.Inadequate attention to CNCSC services** | The ambiguity and undefined roles of CNCSCs in price-setting | Unclear tariffs for preventive activities of centers such as education and counseling |
|  |  |  | The lack of clarity of some duties and the uncertainty of its tariffs |
|  |  |  | They (health system) did not come close to these categories (CNCSCs) therefore, they did not even have that specific definition or tariff. |
|  |  | Delay in updating Irrational and outdated prices | Citation and Criterion of other health provider Centers to determine tariffs of service |
|  |  |  | If tariffs will be real, it's became cost-effective |
|  |  |  | Delayed review of tariffs for services |
|  | **B2.The need to bargain with the CNCSCs and clients to fix a price** | Bilateral (CNCSC-client) efforts for financial profiting | Some Clients demand to change the type of care to reduce costs |
|  |  |  | Clients negotiating to reduce payment costs |
|  |  |  | Suitable condition for extortion financial due to the unclear CNCSCs service tariffs |
|  |  |  | Customer's desire to receive more expensive services in emergency situations |
|  |  |  | Importance of updating tariffs along with paying ability of peoples. |
|  |  | Clients being uncertain about the price of nursing services offered at CNCSCs | The heterogeneity of the proposed tariff for similar services by CNCSCs |
|  |  |  | Customer comparison regarding the cost of paying at the physician office and the cost of paying for CNCSCs services |
|  |  |  | Heterogeneity of tariffs and differences in the prices of services between clinics and CNCSCs for clients |
|  |  |  | Customer's ambiguity due to different payments for similar care services |
|  | **B3. A defective environment of competence** | Independent nursing home care by nurses and clinics | Direct relationship with the recipients at the place of admission by hospital employed nurses |
|  |  |  | Creating a network and an independent team of nurses to provide specialized care services |
|  |  |  | Disconnecting working relationship with the CNCSCs and establishing a direct customer relationship by some careers |
|  |  |  | Failure to comply with work boundaries and provide nursing care at home by clinics |
|  |  | Easier access to inexpensive, although unprofessional and low-quality, home care services | Use of non-specialized services because of cost preferences |
|  |  |  | Failure to recognize centers and individuals with or without permission and incompetent |
|  |  |  | Provide Unprofessional nursing services without equipment and a suitable team |
|  |  |  | Non-specialize managers at non-regulated activities |
|  |  |  | The inability of CNCSCs managers to prevent non-regulated activities. |
|  |  |  | The weakness in audit process and the lack of preventive action over the non-regulated activities |
|  |  |  | Complication and costs of unprofessional care |
|  |  | The client’s preference for receiving services from hospitals and governmental health care centers given their comparatively lower prices | Requests for patient care in the hospital clients without career or with financial problems |
|  |  |  | Peoples low awareness of the CNCSCs activities and capabilities |
|  |  |  | Reducing the level of health care provision in the community due to lower tariffs in public hospitals and clinics |
|  |  |  | Increased demand for patients' housing in hospitals special wards due to the low cost of services |
|  |  |  | Increasing hospital stay and reducing client’s tendency to receiving homecare. |

**Table2. Exploring the challenges in the financial transaction between CNCSCs and their clients**

| **Main Category** | **Subcategory** | **Initial Code** | **Primary(Raw data)** |
| --- | --- | --- | --- |
| C: **The use of benefit strategies for cost-effective services and client satisfaction** | **C1. Regulating financial transactions with the families** | Financial transactions limited to only one of the family members | Trying to encounter and contract with one of family members |
|  |  |  | A financial contract with focus on paying responsibility by one of family members |
|  |  |  | Renewable Contracts by create mutual confidence |
|  |  | Avoiding a direct financial transaction between the CNCSCs staffs and the family members | Monopolize of receiving services costs solely by the CNCSCs |
|  |  |  | The likelihood of getting used to, repeating the mistake of receiving money from clients and creating distrust in view of others |
|  |  |  | Disconnect work relationship with staff how get money from the client |
|  | **C2.** **Expediency trying to expand organizational relationships** | A cautious work relationship with some semi-private organizations | Proposed sub-tariffs by private insurance companies such as banks |
|  |  |  | Low and dispersed customers reason to withdraw from the contract with banks insurance coverage |
|  |  |  | Income for some centers and the loss of other centers in the insurance contract of banks insurance coverage |
|  |  |  | Not suitable tariffs by private insurance, such as banks |
|  |  | Trying to expand professional relationships with rich and financially-independent organizations | The possibility of contracting with large and valid companies for care coverage |
|  |  |  | The cost-effectiveness of contracting with some relatively rich and powerful in permanent organizations with request consistent services for chronic home-patients |
|  |  | Trying to expand the delivery of services to clients with complementary insurance coverage | Having a license provide suitable situation for working contract |
|  |  |  | Contracts and cooperation of CNCSCs with some of health centers and providing part of theirs services |
|  |  |  | CNCSCs working Contract by private hospitals |
|  |  |  | The willingness of home and patient care provided by the patient and family with institutional coverage and support |
|  |  |  | Efforts to expand the coverage and coverage of insured individuals such as the municipality |
|  | **C3.Trying to rationalize the costs of service for the clients** | Informing the clients about the sensitivity and benefit of community-based nursing services | Persuading the family to accept care and costs after providing explanation |
|  |  |  | We justify the family as well, thinking that if you have less than 100.000 IRR, it’s better |
|  |  |  | Tray to notice clients to reduction of indirect and expired services costs |
|  |  | Assisting the clients in preparing adequate documentation for the reimbursement of costs by complementary insurance | Confirmation of the factors to the physician by informal relationship |
|  |  |  | Introducing alternative services in issuing invoices for quick and complete costsrepayment |
